# Assessing Metagenomic Signals Recovered from Lyuba, a 42,000-Year-Old Permafrost-Preserved Woolly Mammoth Calf

**DOI:** 10.3390/genes9090436

**Published:** 2018-08-31

**Authors:** Giada Ferrari, Heidi E. L. Lischer, Judith Neukamm, Enrique Rayo, Nicole Borel, Andreas Pospischil, Frank Rühli, Abigail S. Bouwman, Michael G. Campana

**Affiliations:** 1 Institute of Evolutionary Medicine, University of Zurich, 8057 Zurich, Switzerland; judith.neukamm@iem.uzh.ch (J.N.); enrique.rayo@iem.uzh.ch (E.R.); frank.ruehli@iem.uzh.ch (F.R.); abigail.bouwman@iem.uzh.ch (A.S.B.); 2 Centre for Ecological and Evolutionary Synthesis (CEES), Department of Biosciences, University of Oslo, 0316 Oslo, Norway; 3 Institute of Evolutionary Biology and Environmental Studies, University of Zurich, 8057 Zurich, Switzerland; heidi.lischer@ieu.uzh.ch; 4 Swiss Institute of Bioinformatics (SIB), 1015 Lausanne, Switzerland; 5 Institute for Archaeological Sciences, University of Tübingen, 72070 Tübingen, Germany; 6 Institute of Veterinary Pathology, University of Zurich, 8057 Zurich, Switzerland; n.borel@access.uzh.ch (N.B.); apos@vetpath.uzh.ch (A.P.); 7 Center for Conservation Genomics, Smithsonian Conservation Biology Institute, Washington, DC 20008, USA

**Keywords:** ancient DNA, *Mammuthus primigenius*, microbiome, environmental DNA, DNA contamination

## Abstract

The reconstruction of ancient metagenomes from archaeological material, and their implication in human health and evolution, is one of the most recent advances in paleomicrobiological studies. However, as for all ancient DNA (aDNA) studies, environmental and laboratory contamination need to be specifically addressed. Here we attempted to reconstruct the tissue-specific metagenomes of a 42,000-year-old, permafrost-preserved woolly mammoth calf through shotgun high-throughput sequencing. We analyzed the taxonomic composition of all tissue samples together with environmental and non-template experimental controls and compared them to metagenomes obtained from permafrost and elephant fecal samples. Preliminary results suggested the presence of tissue-specific metagenomic signals. We identified bacterial species that were present in only one experimental sample, absent from controls, and consistent with the nature of the samples. However, we failed to further authenticate any of these signals and conclude that, even when experimental samples are distinct from environmental and laboratory controls, this does not necessarily indicate endogenous presence of ancient host-associated microbiomic signals.

## 1. Introduction

The field of paleomicrobiology has experienced significant advances thanks to the development of high-throughput DNA sequencing techniques: From the first attempts to extract ancient DNA (aDNA) from mummified tissues [1], to validating the presence of pathogens [2,3,4], to reconstructing ancient bacterial genomes [5,6], to recovering entire ancient microbial communities [7,8]. Given the importance of commensal microbes in human health [9,10,11], increasing numbers of microbiome studies have been conducted in recent years. Consequently, much attention has also been given to the taxonomic reconstruction of ancient microbiomes, with a particular focus on human fecal and oral microbiomes using coprolites [12] and dental calculus [7,8,13]. Such approaches offer an ideal opportunity to observe the evolution of these site-specific microbial communities over time.

Since the majority of ancient microbiome studies rely on archaeological material, particular attention needs to be given to the issue of environmental exposure to closely related soil taxa. The presence of DNA from unsequenced or poorly characterized environmental microorganisms potentially, related to pathogens can confound paleomicrobiological studies [14,15]. Such issues are even more prominent when attempting to reconstruct entire ancient microbial communities and authentication procedures need therefore to address environmental signals [16,17]. Additionally, the same issues can be caused by contaminants in laboratory reagents [18,19,20].

Here, we illustrate the issue of environmental and laboratory contamination by attempting to reconstruct tissue-specific ancient metagenomes from a permafrost-preserved woolly mammoth (*Mammuthus primigenius*, Blumenbach 1799) calf. Asian elephants (*Elephas maximus*), the closest living relatives of woolly mammoths [21], are known to be affected by a number of microbial diseases, such as elephant endotheliotropic herpesviruses [22] or tuberculosis [23]. We therefore set out to investigate whether this was the case for woolly mammoths too, and chose a shotgun metagenomics approach, in order to capture the entire microbial diversity.

**Figure 1:**
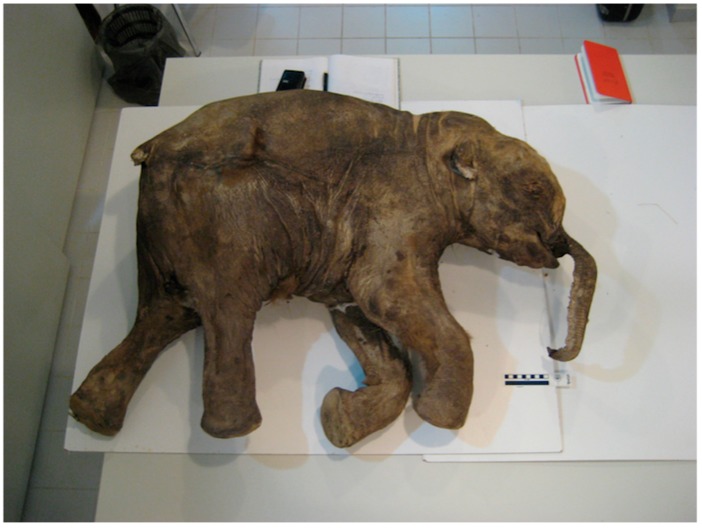
Lateral right view of Lyuba (photo credit: Daniel C. Fisher. Museum of Paleontology, University of Michigan, Ann Arbor, MI, USA). From Reference [27], reprinted by permission of John Wiley & Sons, Inc. (Hoboken, NJ, USA).

The calf, originally named “Lyuba”, was discovered by Nenets reindeer herders in 2007, exposed on a point bar along the Yuribey river on the Yamal peninsula, northwestern Siberia (68°38′ N, 71°40′ E) [24,25]. Lyuba (Figure 1) is the best preserved mummified woolly mammoth ever found, presenting only slight skin lesions [24]. Radioisotopic analyses of bone collagen indicate that it lived approximately 41,800 ^14^C years BP [25], and anatomical investigations revealed that the calf was female and in a good nutritional state [24,25]. The age at death was estimated at 30–35 days, based on a dentin neonatal line and postnatal dentin increments on its deciduous teeth [26]. Vivianite (hydrated iron phosphate), was found in Lyuba’s trachea and bronchi, suggesting the cause of death to be suffocation after inhalation of mud [25]. Due to Lyuba’s accidental cause of death, good health, and excellent preservation, several studies have been performed on its remains, since findings can potentially be extended to juvenile woolly mammoths in general.

Fisher and colleagues [25] argue that the point bar on which Lyuba was found was not likely to represent the site of death or preservation. They conclude that an ice-out flooding during the previous summer is the most likely natural process to have moved the calf to its discovery location. Therefore, Lyuba must have endured at least one cycle of thawing and freezing before discovery, a finding that has also been corroborated by histological analysis [27].

Dissections were performed on Lyuba in 2008 and 2009 [25], and several samples, including intestinal, peritoneal, muscle, and fat tissues, and vivianite, were obtained for further analysis. Here, we attempted to reconstruct these samples’ metagenomes through shotgun high-throughput DNA sequencing. Despite displaying a large component derived from environmental and laboratory contamination, the tissue-specific metagenomes were taxonomically different from non-template and environmental controls. Furthermore, some bacterial species were only associated with one specific tissue, consistent with a potential bacterial infection or a tissue-specific microbiomic signal. However, we failed to further authenticate any of these signals and conclude that, in this case, all detected signals were the result of laboratory or environmental contamination. Caution and strict validation procedures are therefore recommended even under ideal morphological preservation circumstances and when comparison with laboratory and environmental control samples suggest endogenous presence of microbial species.

## 2. Materials and Methods

### 2.1. Samples

The samples used in this study (donated by Daniel C. Fisher, Museum of Paleontology, University of Michigan, 1109 Geddes Ave., Ann Arbor, MI 48109-1079, USA) were obtained during endoscopical examination, and partial dissections performed in 2008 and 2009, and described in References [25,27]. The authors were not present during these procedures, but received the samples at a later stage. This study was conducted with written permission of the sample donors and of the International Mammoth Committee. Lyuba was kept frozen during the time intervals between discovery and the two dissections. The samples used in this study were previously labeled as follows: Abdominal oblique muscle, abdominal subcutaneous fat and muscle, intestinal tissue (two samples), caecum, omentum, peritoneum, cheek fat, and vivianite.

### 2.2. DNA Extraction

The samples’ surfaces were cleaned with a 1% NaOCl solution and UV-irradiated for 10 min to reduce contamination. One hundred milligrams of homogenized tissue were digested at 55 °C for 18 h, followed by three days rotating at room temperature, in 1 mL of extraction buffer (50 mM Tris-HCl, 100 mM NaCl, 25 mM EDTA, 2% SDS and 0.5 mg/mL proteinase K). Following centrifugation, the supernatant was extracted twice with a 25:24:1 phenol, chloroform, and isoamyl alcohol mixture, followed by a final chloroform step. DNA was isolated using QIAquick spin columns (QIAGEN, Hombrechtikon, Switzerland), with two elutions in 30 μL EB buffer and reduced centrifugation speed (6–10 krpm) to prevent loss of short DNA fragments. Extractions were performed in a dedicated aDNA clean laboratory at the University of Zurich [28], which has an independent HEPA (High Efficiency Particulate Air) air filtration system, is physically separated from all laboratories in which PCR is performed, and follows established anti-contamination protocols [29,30], including, unidirectional work flows, overhead UV lights, regular sterilization of all work surfaces, the use of full body suits, masks, and gloves by all researchers, and the parallel processing of non-template controls.

### 2.3. Library Preparation and Sequencing

Ten microliters of extract or extraction blank were converted into double-indexed Illumina sequencing libraries following a protocol optimized for aDNA [31,32]. While all samples had unique index pairs, some shared a single index. Sample-specific indexes were added to each library via 10 cycles of PCR amplification [32]. Blunt-end repair, adapter ligation and set up of indexing PCRs were performed in an aDNA clean laboratory and non-template library blanks were generated in parallel. In order to increase library concentrations to 10^13^ copies/μL, a re-amplification step was performed on all indexed libraries in 100 μL reactions containing 1 unit AccuPrime™ *Pfx* DNA polymerase (Thermo Fisher Scientific, Reinach, Switzerland), 1× AccuPrime™ *Pfx* reaction mix, 0.3 µM primers IS5 and IS6 [31] and 0.5–4 μL library template with the following thermal profile: Initial denaturation at 95 °C for 2 min, 9–18 cycles of denaturation at 95 °C for 15 s, annealing at 60 °C for 30 s and elongation at 68 °C for 1 min, followed by a final elongation at 65 °C for 5 min. Libraries were purified with MinElute spin columns (QIAGEN, Hombrechtikon, Switzerland) following the manufacturer’s instructions. Quantitative PCR and analysis on an Agilent 2100 Bioanalyzer DNA 1000 chip were used to assess library qualities. Libraries were pooled equally and sequenced on a MiSeq (Illumina, San Diego, CA, USA) with paired-end 150 bp reads, as well as on 1.5 lanes of a HiSeq2500 (Illumina) with paired-end 125 bp reads and v4 chemistry by the Functional Genomics Center Zurich (Zurich, Switzerland). While all samples were sequenced on the same MiSeq lane, individuals which shared an index were separated across the two HiSeq lanes to limit index switching [32].

### 2.4. Sequence Quality Control and Filtering

All Illumina paired-end reads (MiSeq and HiSeq) were processed using the same pipelines. Raw reads were demultiplexed using DeML [33] with default settings. Due to low-quality p5 sequences, the HiSeq data were demultiplexed using only the p7 adapter sequence. Trimmomatic 0.33 [34] was used to remove adapter sequences and artifacts with the following parameters: Maximum seed mismatches = 2, palindrome clip threshold = 30, simple clip threshold = 7, minimum adapter length = 5, and retaining reverse reads. Leading and trailing bases below Phred quality score 3 were removed, reads were scanned using 4 bp sliding windows and trimmed when average Phred quality score fell below 15. Trimmed reads shorter than 25 bp were discarded. Surviving paired reads were merged with SeqPrep [35] as described in Reference [36] to increase sequence quality. Merged reads shorter than 25 bp and unmerged reads were discarded. Finally reads qualities were assessed using FastQC 0.10.1 [37].

### 2.5. Mapping to Reference Genomes and Deamination Pattern Analysis

Reads were mapped to the African elephant (*Loxodonta africana*) nuclear genome (NW_003573420.1), the woolly mammoth (*M. primigenius*) mitochondrial genome (EU155210.1), and the human reference genome (GRCh38.p2) using the Burrows-Wheeler Aligner (BWA) 0.7.12 aln algorithm [38], with disabled seeding, increased gap open (-o 2), and reduced edit distance (-i 0) as recommended in Reference [39]. Duplicates were removed with Picard 2.1.0 [40] with validation stringency set to lenient, and filtering for a minimal mapping quality of 25 was performed with SAMtools 1.3.1 [41]. DNA deamination patterns of mapped reads were analyzed with mapDamage2 2.0.6 [42].

### 2.6. Mitogenome Analysis

MiSeq and HiSeq reads, that mapped to the woolly mammoth mitochondrial genome for all samples, excluding vivianite, were merged into a single BAM file, duplicates were marked as described above and a consensus sequence was built using SAMtools 1.3.1 [41]. LASTZ [43] was used to compare the obtained consensus sequence with the Lyuba mitochondrial genome published by Enk and colleagues [44] (KX027526.1).

### 2.7. Shotgun Metagenomic Analysis

From this step onwards, published Illumina shotgun metagenomes from two Asian elephant (*E. maximus*) fecal samples (SRP040073) [45] and two Russian permafrost samples (SRP049520) [46] obtained from the Sequence Read Archive were included. The elephant fecal samples were obtained from three-week-old and six-year-old Asian elephants (*E. maximus*). No other comparable elephant metagenomic datasets are currently available. The permafrost samples originated from lake sediment (alluvium) from the Panteleikha River floodplain and from the late Pleistocene Ice Complex on the Omolon River, both located in northeast Siberia. These reads were processed using the same parameters as for the Lyuba samples. Quality-controlled, merged reads from the libraries generated here and the reference data were deduplicated by clustering and removing all exact matches using CD-HIT-EST 4.6 [47]. Deduplicated reads were compared to the National Center for Biotechnology Information (NCBI) nucleotide collection (downloaded January 2018) using the megablast algorithm (BLAST 2.6.0+ [48]) with default parameters. To verify megablast species identifications, we additionally analyzed the Lyuba, vivianite, and laboratory controls with MALT [49] using a curated reference database consisting of 5242 complete bacterial genomes available in NCBI RefSeq (December 2015). The BlastN algorithm with SemiGlobal alignment was used. Megablast and MALT results were visualized with MEGAN6 (builds 6.11.6 or 6.10.6, respectively) [50]. The megablast results were analyzed using MEGAN default Lowest Common Ancestor (LCA) parameters, while the MALT results used the following options: --topPercent 1.0, --minSupportPercent 0, and --minSupport 5. Sample comparisons used normalized counts to control for variation in sequencing depth.

### 2.8. 16S Metagenomic Analysis

16S metagenomic analysis was performed using the QIIME2 (version 2018.4) [51] package and its associated plugins. All analyses were performed using default settings unless otherwise specified. Deduplicated reads from the novel and published shotgun libraries were closed-reference clustered at 99% sequence identity to the SILVA database release 132 [52] using VSEARCH [53]. Retained sequences were aligned and a phylogenetic tree was constructed using MAFFT [54] and FastTree [55]. The library blank was excluded from diversity metric calculations because only nine sequences were retained after clustering. Phylogenetic and non-phylogenetic diversity metrics were calculated with rarefication to 149 and 945 sequences, corresponding to the next two smallest sample sizes (extraction blank and intestinal tissue 2, respectively) from our novel Lyuba dataset. Principal Coordinate Analyses of Bray-Curtis, Jaccard, and Unweighted UniFrac distances were visualized using EMPeror [56,57].

Clustered sequences were classified according to the SILVA consensus taxonomy (all levels) using VSEARCH [53] (99% nucleotide identity). Clustered sequences were exported from QIIME 2 and analyzed using SourceTracker 2 [58]. All mammoth tissue samples were classified as ‘sinks’, while the permafrost, blanks, feces and vivianite samples were treated as ‘sources’. Due to the very small sample sizes, we did not rarefy the sources and rarefied the sinks to 100. All other parameters used their default values.

### 2.9. MetaPhlAn2 Analysis

We also analyzed the deduplicated metagenomes using MetaPhlAn2 (version 2.7.7) under default settings [59]. We generated heat maps clustering the individuals and observed species-level taxa using Euclidean distances.

### 2.10. Authentication of Bacterial Reads

In order to test the authenticity of overrepresented bacterial species in the omentum and cheek fat samples, reads were mapped against the reference genomes of several *Yersinia* species for the omentum sample (*Y. aldovae*, NZ_CP009781.1; *Y. aleksiciae*, NZ_CP011975.1; *Y. enterocolitica*, NC_008800.1; *Y. entomophaga*, NZ_CP010029.1; *Y. fredericksenii*, NZ_CP009364.1; *Y. intermedia*, NZ_CP009801.1; *Y. kristensenii*, NZ_CP008955.1; *Y. pestis*, NC_003143.1; *Y. pseudotuberculosis*, NZ_CP008943.1; *Y. rohdei*, NZ_CP009787.1; *Y. ruckeri*, NZ_CP011078.1; and *Y. similis* NZ_CP007230.1), as well as to the *Alcaligenes faecalis* reference genome (NZ_CP013119.1) and the *Carnobacterium* sp. CP1 genome (NZ_CP010796.1) for the cheek fat sample, using the same parameters as described above. DNA deamination patterns of mapped reads were analyzed with mapDamage2 2.0.6 [42].

### 2.11. Data Availability

Raw sequencing data have been deposited in the NCBI Sequence Read Archive (accession number SRP113695).

## 3. Results

### 3.1. Data Quality Control and Authentication

**Figure 2:**
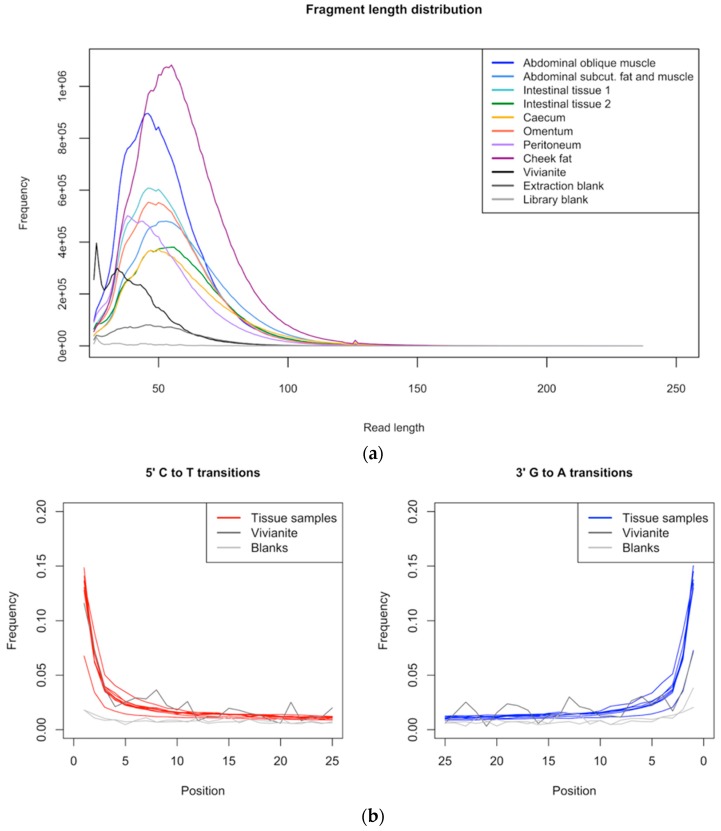
Ancient DNA (aDNA) fragmentation and misincorporation patterns. (**a**) Read length distribution of quality-filtered reads is shown for all samples and controls. Average read length ranges from 34 to 56 bp. (**b**) Quality-filtered reads were mapped to the African elephant nuclear genome with a minimum mapping quality of 25 and nucleotide misincorporation rates were calculated using mapDamage. Increased cytosine deamination rates at 5′-overhangs are visible (C to T and G to A transitions), consistent with aDNA.

We obtained 17–47 million reads per sample and, with the exception of the vivianite sample (39% reads surviving), 81–93% of the raw reads survived quality filtering and merging (Table 1). Average read lengths ranged from 34 to 56 bp, with only few reads longer than 100 bp (Figure 2), consistent with fragmentation typical for aDNA [60].

Quality-filtered, merged reads were mapped to the African elephant (*L. africana*) nuclear genome and the human reference genome, as well as the woolly mammoth (*M. primigenius*) mitogenome. Tissue samples showed a highly variable number of reads mapping to the elephant and mammoth references (2.1–43% and 0.1–0.8%, respectively), indicating different levels of endogenous mammoth DNA preservation depending on the tissue of origin (Table 1). The abdominal oblique muscle and cheek fat samples yielded the highest proportions of reads mapping to the elephant nuclear genome (42–43% of total unique reads), whereas for the vivianite sample less than 2500 reads mapped to the elephant nuclear genome (0.28% of total unique reads). Since the vivianite sample did not differ significantly from negative controls in terms of raw data quality and endogenous DNA content, we treated it as an environmental control sample. mapDamage analysis of reads mapped to the African elephant nuclear genome (Figure 2) and woolly mammoth mitochondrial genome (Figure S1a) yielded DNA damage patterns consistent with authentic aDNA, showing increased cytosine deamination rates at 5′-overhangs for all tissue samples [42].

We also observed a low degree of sample contamination with human DNA (0.11–0.81%, Table 1). DNA damage patterns showed a slight increase in cytosine deamination rates at 5′-overhangs (Figure S1b), suggesting that contamination with human DNA happened prior to this study and is consistent with the extensive handling of Lyuba at discovery and recovery. We detected small numbers of unique reads that matched elephant, mammoth and humans in the library and extraction blanks. Since some samples shared indexes with the blanks, these likely derive from sample mis-assignment due to index switching [32].

In order to further validate the authenticity of the sequencing data, we pooled all reads mapping to the woolly mammoth mitogenome together, remapped them to the reference, and built a consensus sequence for the mitogenome of Lyuba. We obtained a sequence with an average per-base coverage of 227×, which was consistent with the previously published Lyuba mitogenome [44].

### 3.2. Shotgun Metagenomic Analysis

In order to reconstruct the taxonomic composition of Lyuba’s tissue-specific metagenomes, we compared quality-filtered, deduplicated reads to the NCBI nucleotide database with megablast and visualized the results with MEGAN6 [50]. Additionally, we included published metagenomes obtained from Asian elephant fecal samples [45] and Russian permafrost sediments [46] for comparison.

**Figure 3:**
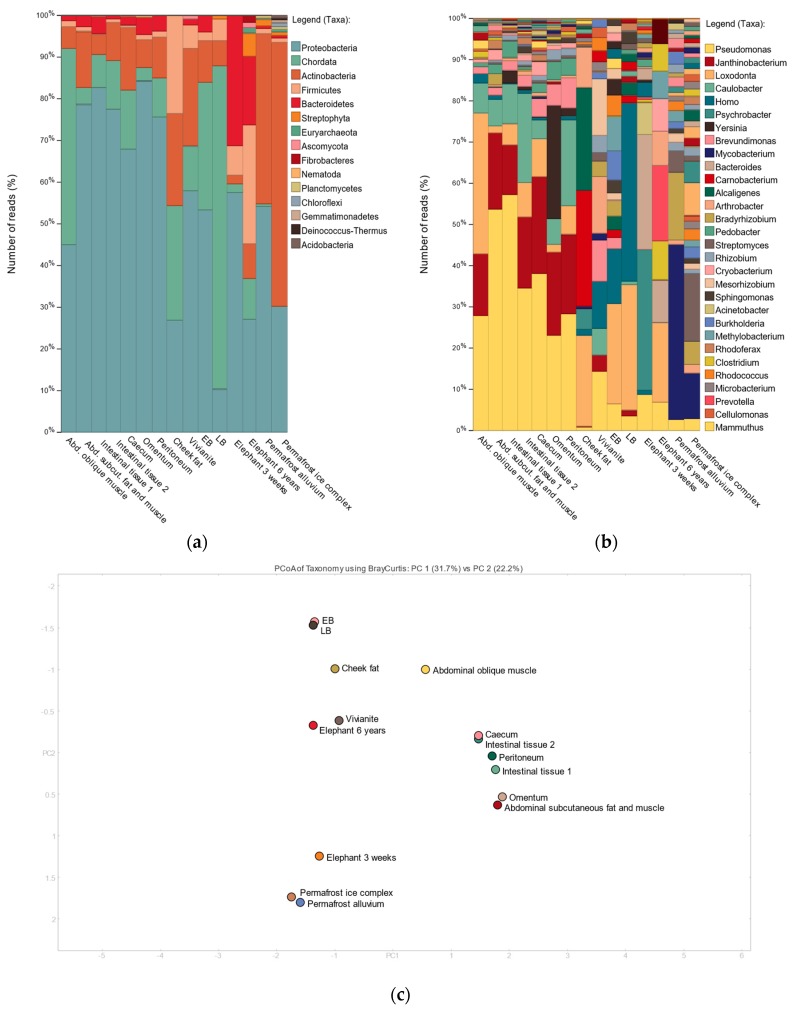
Taxonomic composition of tissue-specific metagenomes. Quality-filtered, deduplicated data were compared to the National Center for Biotechnology Information (NCBI) nucleotide collection with megablast and results were visualized with MEGAN6. Taxa are displayed at the phylum (**a**) and genus (**b**) level. The presence of *Yersinia* in the omentum sample and of *Carnobacterium* and *Alcaligenes* in the cheek fat sample characterize these tissues. For visualization purposes only the most abundant taxa are listed. (**c**) The Bray-Curtis Principal Coordinate Analysis (PCoA) of taxonomic profiles ranked by species shows samples to differ in their taxonomic composition from the non-template controls and the vivianite environmental control, as well as from the permafrost and elephant fecal samples. EB = extraction blank; LB = library blank.

For all Lyuba tissue samples, reads could be assigned to the genera *Loxodonta*, *Elephas*, or *Mammuthus* (25,000–600,000 reads per sample), with the abdominal oblique muscle sample yielding the most reads (Figure 3b). Furthermore, as expected for this kind of material, we observed a large environmental DNA component in Lyuba’s tissues, with the most commonly occurring bacterial species being known environmental, soil- or water-dwelling, bacteria (Figure 3a,b). For example, roughly 50-80% of the metagenome of all tissue samples, excluding the cheek fat sample, is composed of bacterial species belonging to the genera *Pseudomonas*, *Janthinobacterium*, *Caulobacter*, and *Brevundimonas*. All these taxa are present in the vivianite control, and in at least one of the laboratory non-template controls, suggesting contamination from laboratory reagents or workflows, the environment, or both. Despite this, the remaining fraction of the tissue samples’ metagenomes appear to have overall different taxonomic compositions and to differ from the non-template controls as well as from the vivianite control (Figure 3b). This can also be seen in the Principal Coordinate Analysis (PCoA) of taxonomic profiles at the species level (Figure 3c), where the tissue samples cluster separately from the laboratory and vivianite controls and the cheek fat sample is most distant from the other tissue samples. Contrary to expectations, while mammoth samples of intestinal origin cluster together, they are not taxonomically similar to the elephant fecal samples. Similarly, the vivianite sample is taxonomically different from the permafrost samples.

Interestingly, we also observed a few bacterial taxa that were particularly abundant in the metagenome of one of Lyuba’s tissue samples and nearly or completely absent from the other tissues and controls. In particular, we detected the presence of *Yersinia* (354,641 summed reads) in the omentum sample and of *Carnobacterium* (307,572 summed reads) and *Alcaligenes* (272,401 summed reads) in the cheek fat sample. While species of the genus *Yersinia* are widely found in the environment, mostly in fresh water and soil, some are important pathogens for humans and other animals (*Y. pestis*, *Y. pseudotuberculosis* and *Y. enterocolitica*) and yet others are capable of opportunistic infections [61]. The majority of reads (330,049) were assigned at the genus level rather than to one or more species in particular. Similarly, *A. faecalis*, to which the majority of the *Alcaligenes* reads (271,878) were assigned to, is a common soil bacterium and human opportunistic pathogen [62]. In contrast, there are no known pathogens in the genus *Carnobacterium* [63]. However, its presence in the cheek fat sample is interesting, since acidification of Lyuba’s tissues through lactic-acid-producing bacteria has been suggested as an explanation for its exceptional preservation and lack of scavenging during the time the calf was exposed between the ice-out flooding and its recovery [25]. The majority of reads (201,132) were assigned to *Carnobacterium* sp. CP1.

When ranking bacterial taxa in Lyuba’s tissues at the genus and species level and ignoring all taxa that were present in the laboratory or environmental vivianite controls (with a minimum of 50 assigned reads), we observed a much lower diversity in taxonomic composition (Figure S2). Furthermore, all detected species and genera are common soil- or water-dwelling bacteria. The only exception is the marked presence of *Yersinia* in the omentum sample. Because the laboratory controls contained 80–160 reads that were assigned to *Alcaligenes* or *Carnobacterium*, we could not observe these taxa in the cheek fat sample in this analysis. However, given the high number of reads in the tissue sample (272,401 and 307,572 summed reads, respectively), this could also be due to index switching rather than to laboratory contamination.

Finally, we also analyzed all quality-filtered reads obtained from Lyuba’s tissue samples and controls with MALT [49] and visualized the results with MEGAN6 [50]. Results were generally consistent with the outcome of the megablast analysis (Figure S3), but we did observe some discrepancies. For example, the MALT analysis did not detect *Janthinobacterium* as a major component of most of Lyuba’s tissues. We also obtained fewer reads assigned to the genus *Yersinia* (95,021 summed reads) in the omentum sample. Furthermore, we did not observe the presence of *A. faecalis* in the cheek fat sample. These discrepancies are due to the fact that the reference database we utilized for the MALT analysis contains fewer *Janthinobacterium* and *Yersinia* spp. genomes and lacks the *A. faecalis* genome.

### 3.3. 16S Metagenomic Analysis

**Figure 4:**
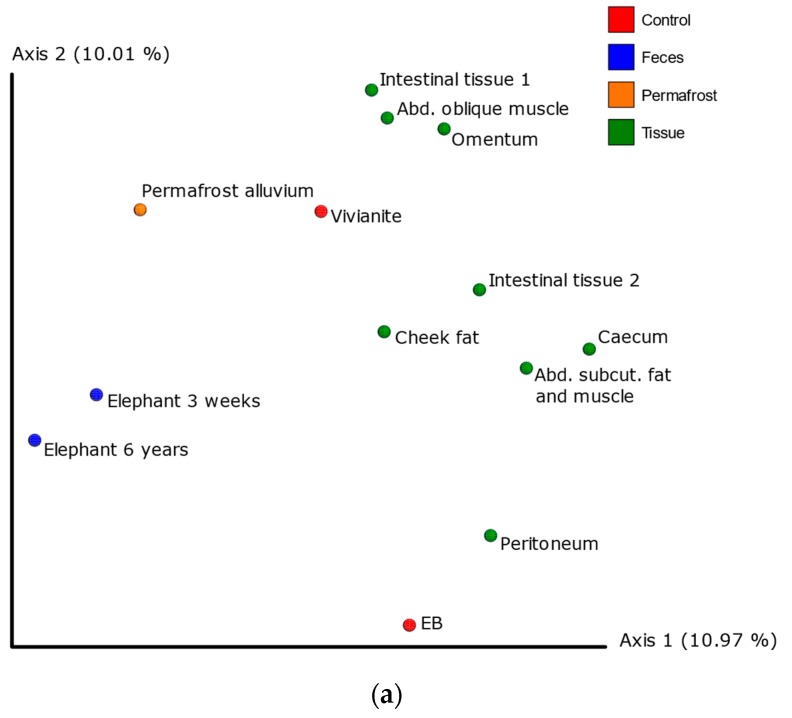

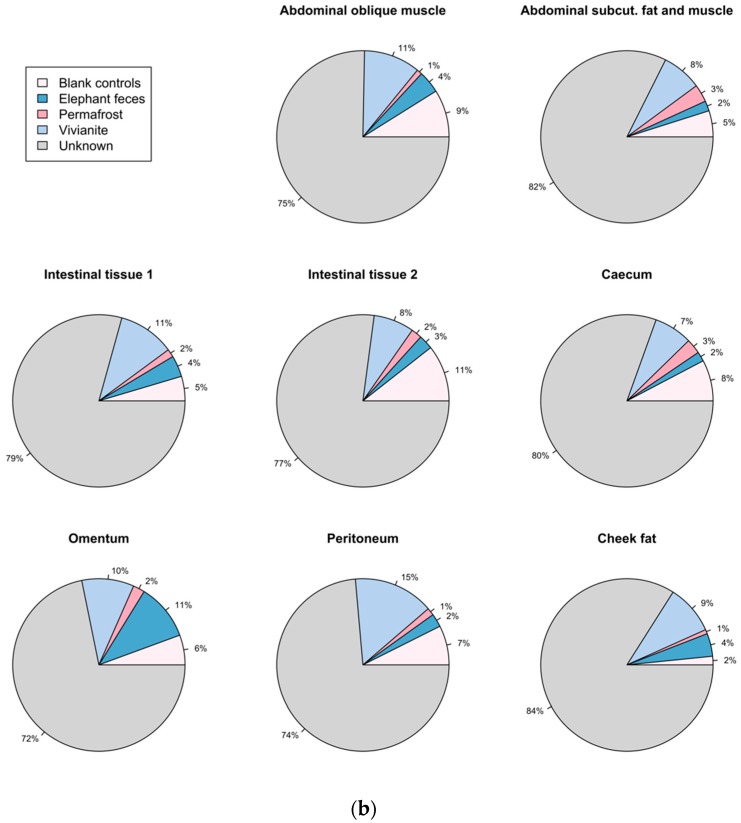
QIIME2 16S metagenomic analysis. (**a**) PCoA of Unweighted UniFrac distances. Phylogenetic diversity metrics were calculated with rarefication to 149 sequences. (**b**) SourceTracker analysis. Laboratory blank controls, the vivianite environmental control, the elephant fecal samples, and the Russian permafrost samples were set as possible sources of microbial communities.

The 16S metagenomic analysis retained between 9 (library blank) and 8037 (elephant 3 weeks) unique sequences (mean: 1739 sequences per sample; standard deviation: 1988 sequences) after clustering to the SILVA database. We identified *Yersinia* in the omentum sample at a 0.84% relative frequency and *Alcaligenes* and *Carnobacterium* in the cheek fat sample at 1.77% and 1.56% relative frequencies, respectively (Figure S4). All PCoA analyses produced concordant results: With the exception of the cheek fat sample, the Lyuba tissues clustered with themselves near the controls (Figure 4a and Figure S5). The cheek fat sample was the most similar to the elephant feces. The tissue samples did not cluster with the permafrost sample. These results are concordant with those of the SourceTracker analysis (Figure 4b): ~10% of tissue reads derived from the local environment (represented by vivianite) and ~5% derive from laboratory contamination. The cheek fat sample had the lowest laboratory contamination level (2%) which may explain its differentiation in the PCoAs. Interestingly, the omentum had the highest proportion of reads that correspond to the elephant fecal microbiome (11%), but still clustered with the other Lyuba tissue samples.

### 3.4. MetaPhlAn2 Analysis

The MetaPhlAn2 analysis results largely replicated the results of the megablast and MALT analyses (Figure S6). With the exception of the cheek fat sample, the Lyuba tissue metagenomes were dominated by *Pseudomonas*, *Janthinobacterium*, *Caulobacter*, *Pedobacter*, and *Brevundimonas*. *Yersinia intermedia* was detected in the omentum and intestinal tissue 1 sample. *Alcaligenes*, *Carnobacterium* sp. 17.4, and Arthrobacter gangostriensis were found in the cheek fat. Only *Brevundimonas* was identified in the vivianite. With the exception of the cheek fat, the Lyuba tissue samples cluster together as in the other analyses.

### 3.5. Authentication of Potential Ancient Bacterial Signals

While the taxonomic composition of Lyuba’s tissue-specific metagenomes was overall similar, the presence of *Yersinia*, *Carnobacterium* sp. CP1, and *A. faecalis* as prominent components in the omentum and cheek fat samples’ metagenomes did set these two tissues apart. In order to test whether the presence of these bacteria represented authentic ancient host-associated microbiomic signatures, we mapped all reads obtained from the omentum sample against the reference genomes of various *Yersinia* species, and the reads obtained from the cheek fat sample to the *A. faecalis* reference genome and the *Carnobacterium* sp. CP1 genome. We then analyzed the DNA damage patterns of all mapped reads to test whether we could detect typical aDNA deamination.

**Figure 5:**
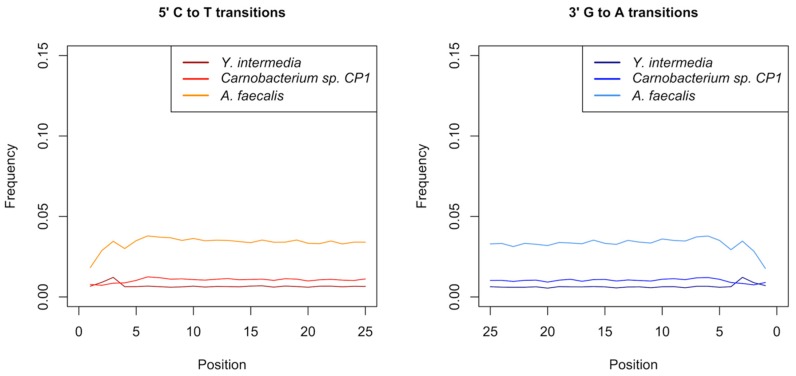
DNA damage pattern analysis of omentum reads mapped to *Y. intermedia* and cheek fat reads mapped to *Carnobacterium* sp. CP1 and *Alcaligenes faecalis*. Quality-filtered reads were mapped to the reference genomes with a minimum mapping quality of 25 and DNA damage plots were generated using mapDamage. No increase in cytosine deamination rates at 5′-overhangs is visible (C to T and G to A transitions), which is inconsistent with aDNA.

For the omentum sample we obtained the most reads when mapping against the reference genome of *Y. intermedia* (751,425 reads mapping with a quality of 25 or higher, 10.9% of unique reads). While *Y. intermedia* has been associated with opportunistic gastrointestinal infections, this bacterium is widely found in the environment, mostly in fresh water [61]. Furthermore, a mapDamage analysis showed no increase of cytosine deamination rates at 5′-overhangs (Figure 5), which is inconsistent with authentic aDNA [42].

Similarly, while we obtained 519,810 reads (4.7% of unique reads) mapping to the *Carnobacterium* reference genome and 549,975 reads (5% of unique reads) mapping to the *A. faecalis* reference genome (with a quality of 25 or higher) we failed to detect DNA damage patterns consistent with aDNA (Figure 5).

## 4. Discussion

Ancient microbiome studies are of great value in the context of human health and evolution. However, microbial communities reconstructed from archaeological material typically have a large environmental component. This can lead to false positive signals [14,15] and strict validation procedures are necessary. Additionally, the introduction of microbial contaminants via laboratory reagents also needs to be considered [18,19,20]. Excellent reviews and guidelines on these issues have already been provided by others (e.g., References [16,17]). Based on these guidelines, we attempted to reconstruct the tissue-specific metagenomes of Lyuba, a 42,000-year-old, permafrost-preserved woolly mammoth calf, together with environmental and non-template experimental controls and compared them to metagenomes obtained from permafrost and elephant fecal samples. The endogenous mammoth DNA content was very variable across samples (2–43%), but we obtained DNA deamination patterns consistent with authentic aDNA for all tissue samples [42]. Furthermore, the mitogenome consensus sequence we obtained was consistent with the previously published Lyuba mitogenome [44].

A preliminary taxonomic composition analysis suggested that, despite a large component derived from environmental or laboratory contamination, metagenomes reconstructed from Lyuba’s tissue samples were distinct from those obtained from controls. We paid particular attention to the presence of *Yersinia* in the omentum sample, and of *A. faecalis* and *Carnobacterium* sp. CP1 in the cheek fat sample. These taxa were absent or nearly absent from all other tissues and from laboratory and environmental controls. Therefore, the detection of several hundred thousand of reads in one particular tissue in an otherwise rather uniform taxonomic background, led us to think that these could represent ancient microbiomic signals originating, for example, from an infection in Lyuba’s tissues. Further corroborating this hypothesis, several *Yersinia* species are known pathogens or are at least capable of opportunistic infections [61]. *A. faecalis* is an opportunistic pathogen as well [62] and while there are no known pathogens in the genus *Carnobacterium* [63], the presence of this lactic-acid-producing bacterium is consistent with the proposed explanation that acidification of Lyuba’s tissues contributed to its exceptional preservation [25].

However, these hypotheses were not corroborated by aDNA authentication criteria. In order to identify which *Yersinia* species was most likely to be present in the omentum sample, we mapped all reads against several *Yersinia* reference genomes and obtained the most reads for *Y. intermedia*, a species that is widely distributed in the environment [61]. Additionally, we analyzed deamination rates at 5′-overhangs of omentum reads mapped to *Y. intermedia*, as well as of cheek fat reads mapped to *A. faecalis* and *Carnobacterium* sp. CP1 and found no increase of cytosine deamination, which is inconsistent with authentic aDNA [42]. For these reasons, we cannot conclude that the presence of these bacteria in the omentum and cheek fat samples represents authentic ancient microbiomic signals. We believe that these signals are much more likely to have originated from contamination instead.

We observed several bacterial taxa that were present in almost all of Lyuba’s tissues as well as in one or more of the laboratory and vivianite environmental controls (e.g., *Pseudomonas*, *Janthinobacterium*, *Caulobacter*, and *Brevundimonas*). In this case, the source of contamination can easily be attributed to the environment or to laboratory reagents or workflows. The 16S metagenomic and SourceTracker analyses indicate that our results are affected by these contaminants. However, *Yersinia*, *A. faecalis*, and *Carnobacterium* sp. CP1 were unique to the omentum and cheek fat samples, respectively, with the exception of very few reads that can be explained by index switching. While environmental or laboratory contamination cannot be excluded, they are less likely. Other possible sources of contamination that consider the tissue specificity we observed are contamination during the sampling procedure or a non-uniform colonization of Lyuba’s tissues by environmental bacteria. Lyuba likely endured at least one cycle of thawing and freezing before discovery [25,27]. This may have affected DNA recovery and the fact that we observed very variable amounts of endogenous DNA in the different tissues could reflect variations in DNA preservation. Furthermore, thawing may have offered an opportunity for colonization of Lyuba’s tissues by environmental microorganisms, and if this process did not take place in a homogenous way, it could explain different contaminants among tissues. Moreover, part of the investigated tissues, such as fat and muscle, should be free of bacterial signals, which also indicates post-mortem contamination as a source of the detected bacterial signals.

We conclude that, even in ideal morphological preservation circumstances, host-endogenous microbiome signals can be swamped out by contaminating signals. Therefore, microbiome analyses from ancient tissues must be done with the utmost care, because even when environmental and laboratory controls are not compositionally similar to experimental samples, this does not indicate that microbes are necessarily endogenous.

## Figures and Tables

**Table 1:** Quality filtering and mapping statistics for reads obtained from shotgun sequencing.

	Raw Read Pairs	Merged Reads (% of Total)	Unique Reads (Clonality)	Unique Mapped Reads (% of Unique Reads)		
				*Loxodonta africana*	*Mammuthus primigenius*	*Homo sapiens*
				Nuclear Genome	Mitogenome	
**Abdominal**	35,511,605	31,176,532	102,34,165 (3.05×)	4,371,482	55532	78047
**oblique muscle**		−87.80%		−42.71%	−0.54%	−0.76%
**Abd. subcut.**	22,977,668	21,176,646	6,834,930 (3.10×)	143221	16216	20252
**fat and muscle**		−92.20%		−2.10%	−0.24%	−0.29%
**Intestinal**	25,053,246	23,305,474	7,496,017 (3.11×)	605598	32548	16223
**tissue 1**		−93.00%		−8.08%	−0.43%	−0.22%
**Intestinal**	21,130,716	17,066,995	2,829,012 (6.03×)	270976	12162	6882
**tissue 2**		−80.80%		−9.58%	−0.43%	−0.24%
**Caecum**	17,386,802	16,168,853	5,819,269 (2.78×)	634528	46103	12498
		−93.00%		−10.90%	−0.79%	−0.21%
**Omentum**	22,924,111	21,390,754	6,909,388 (3.10×)	186132	9453	7581
		−93.30%		−2.69%	−0.14%	−0.11%
**Peritoneum**	18,925,818	17,416,113	5,994,320 (2.91×)	405067	6093	14650
		−92.00%		−6.76%	−0.10%	−0.24%
**Cheek**	47,716,128	42,628,440	11,029,081 (3.87×)	1947669	8582	37331
**fat**		−89.30%		−42.08%	−0.19%	−0.81%
**Vivianite**	21,888,799	8,481,750	824,368 (10.29×)	2334	194	6298
		−38.80%		−0.28%	−0.02%	−0.76%
**Extraction**	7,036,101	3,224,382	711,189 (4.53×)	22774	227	5038
**blank**		−45.80%		−3.20%	−0.03%	−0.71%
**Library**	4,820,063	285,265	33387 (8.54×)	8417	151	4479
**blank**		−5.90%		−25.21%	−0.45%	−13.42%

## Supplementary Materials

The following are available online at http://www.mdpi.com/2073-4425/9/9/436/s1, Figure S1: DNA damage pattern analysis of reads mapped to the woolly mammoth mitogenome and the human reference genome, Figure S2: Taxonomic composition of Lyuba’s tissue-specific metagenomes., Figure S3: MALT analysis., Figure S4: QIIME2 16S metagenomic analysis, Figure S5: QIIME2 16S Principal Coordinate Analyses, Figure S6: Heat map of MetaPhlAn2 results based on Euclidean distances.
