# Supplementary material for: Assessing Metagenomic Signals Recovered from Lyuba, a 42,000-Year-Old Permafrost-Preserved Woolly Mammoth Calf
Source: Genes (Basel). 2018 Aug 31;9(9):436. doi: 10.3390/genes9090436 (PMC6162753; doi:10.3390/genes9090436)
Supplement: Supplementary file 1 [file genes-09-00436-s001.pdf]

Supplementary Materials

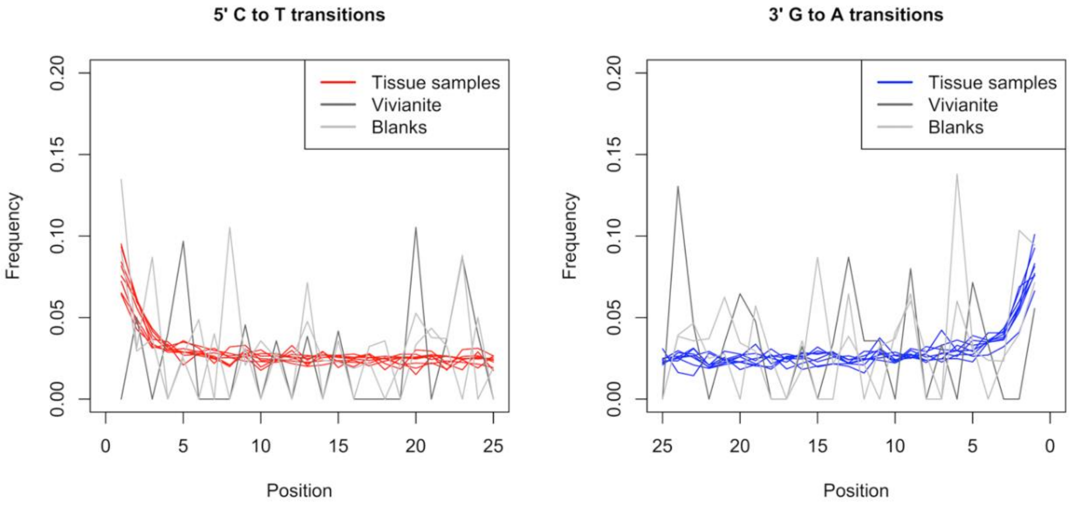

(a)

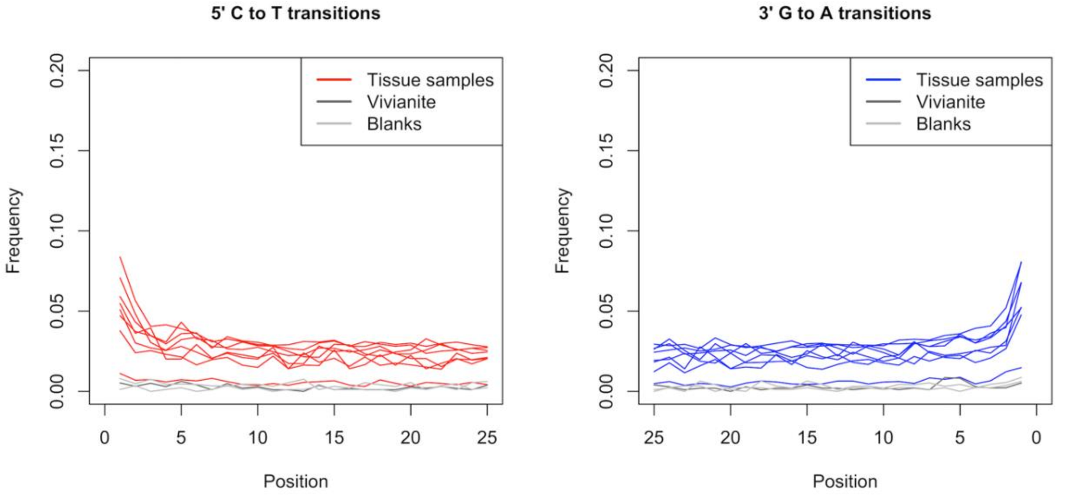

(b)

Figure S1. DNA damage pattern analysis of reads mapped to the woolly mammoth mitogenome and the human reference genome. (**a**) Quality-filtered reads were mapped to the woolly mammoth mitogenome with a minimum mapping quality of 25 and nucleotide misincorporation rates were calculated using mapDamage. Increased cytosine deamination rates at 5′-overhangs are visible (C to T and G to A transitions), consistent with aDNA. (**b**) Similarly, DNA damage patterns were obtained for reads mapping to the human reference genome. The increased misincorporation rates are consistent with a possible human contamination of the samples that occurred prior to this study.

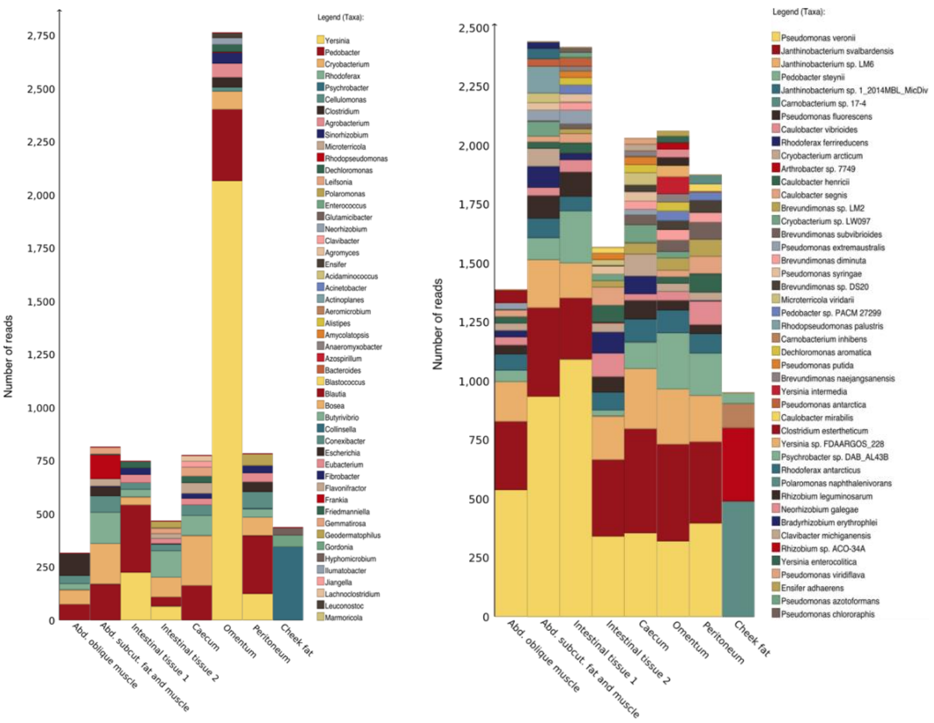

(**a**) (**b**)

Figure S2. Taxonomic composition of Lyuba's tissue-specific metagenomes. Quality-filtered, deduplicated data were compared to the NCBI nucleotide collection with megablast, and results were visualized with MEGAN6. Taxa are displayed at the genus (**a**) and species (**b**) level. Only bacterial taxa are shown. Species or genera occurring in the laboratory and environmental vivianite controls (with a minimum of 50 assigned reads) were ignored. The presence of *Yersinia* in the omentum sample characterizes this tissue. For visualization purposes only the most abundant taxa are listed.

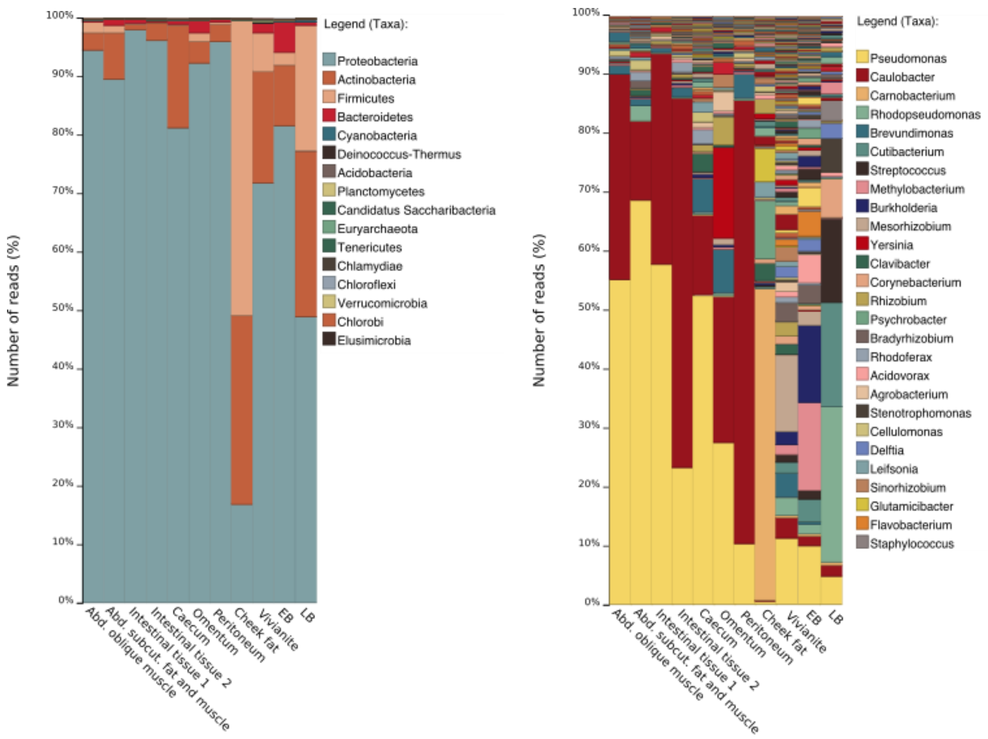

(**a**) (**b**)

Figure S3. MALT analysis. Quality-filtered, deduplicated data were analyzed with MALT and results were visualized with MEGAN6. Taxa are displayed at the phylum (**a**) and genus (**b**) level. *Pseudomonas* and *Caulobacter* make up the majority of the metagenome of all tissue samples, excluding

the cheek fat sample. All tissue samples metagenomes differ in their taxonomic composition from the controls. The presence of *Yersinia* in the omentum sample and of *Carnobacterium* in the cheek fat sample characterize these tissues. For visualization purposes only the most abundant taxa are listed. EB = extraction blank; LB = library blank.

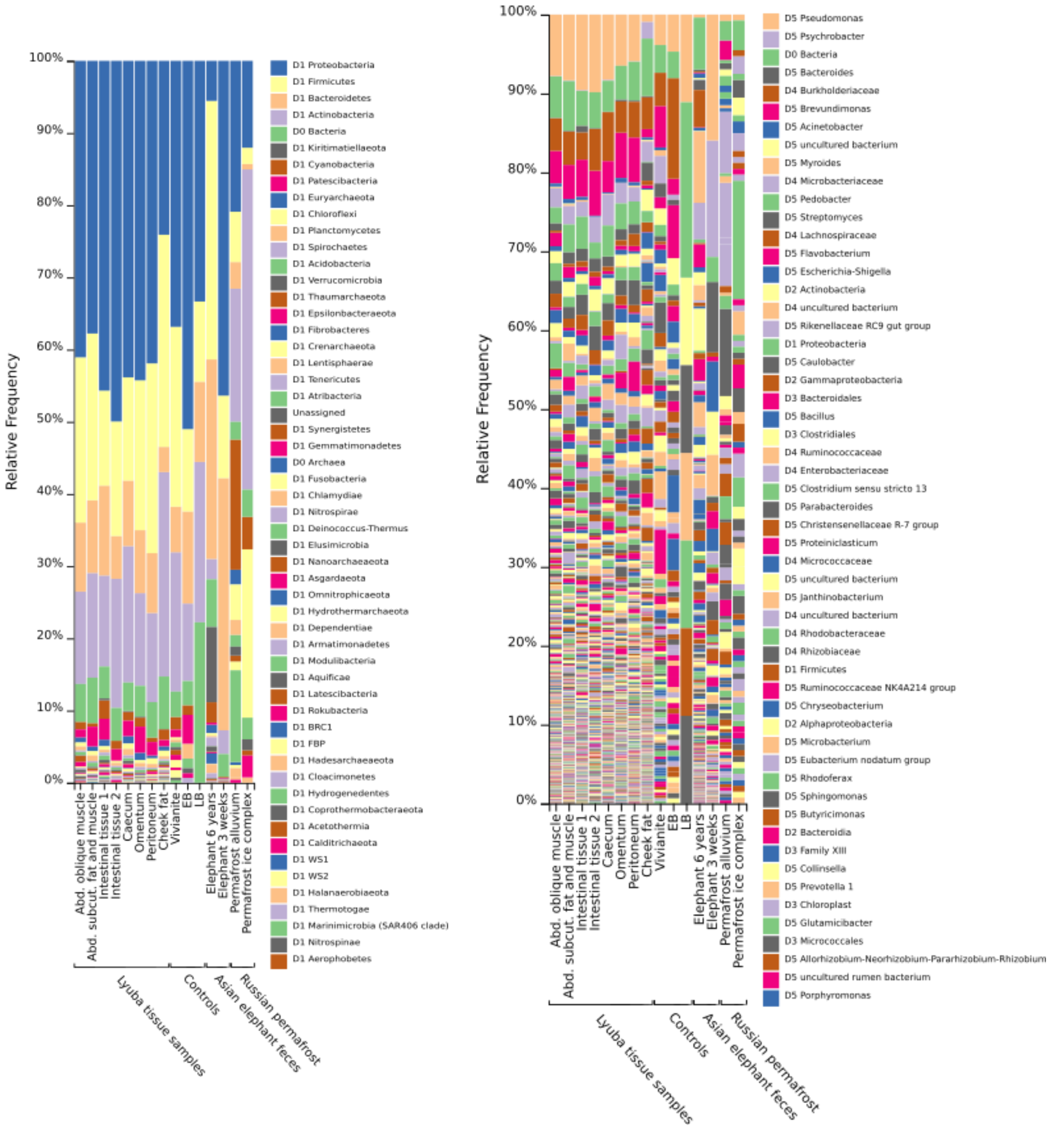

(**a**) (**b**)

Figure S4. QIIME2 16S metagenomic analysis. Clustered sequences were classified according to the SILVA consensus taxonomy. Taxa are displayed at the phylum (**a**) and genus (**b**) level. For visualization purposes only the most abundant taxa are listed. EB = extraction blank; LB = library blank.

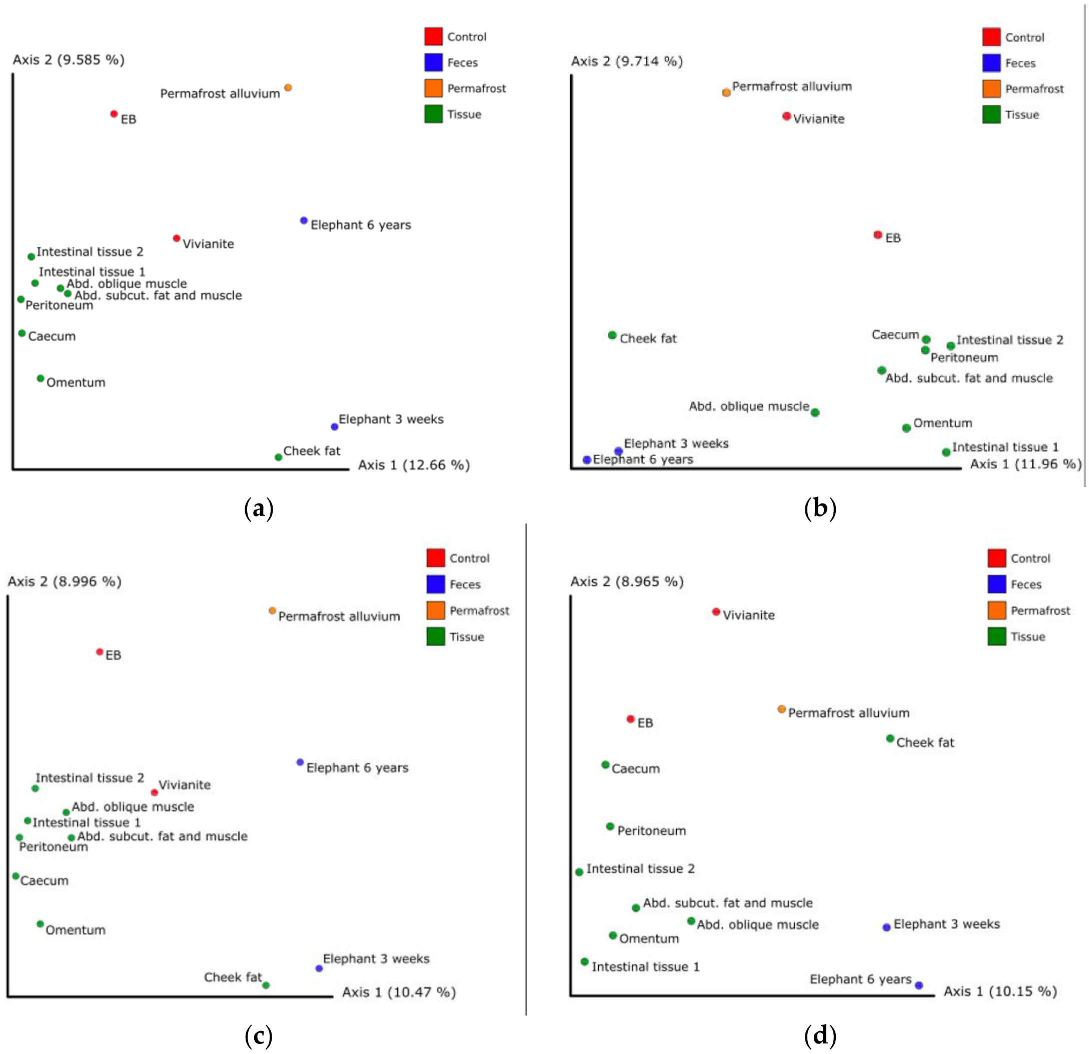

Figure S5. QIIME2 16S Principal Coordinate Analyses. All diversity metrics were calculated with rarefication to 149 sequences. (**a**) Bray-Curtis PCoA with phylogenetic diversity metrics. (**b**) Bray-Curtis PCoA with non-phylogenetic diversity metrics. (**c**) Jaccard PCoA with phylogenetic diversity metrics. (**d**) Jaccard PCoA with non-phylogenetic diversity metrics.

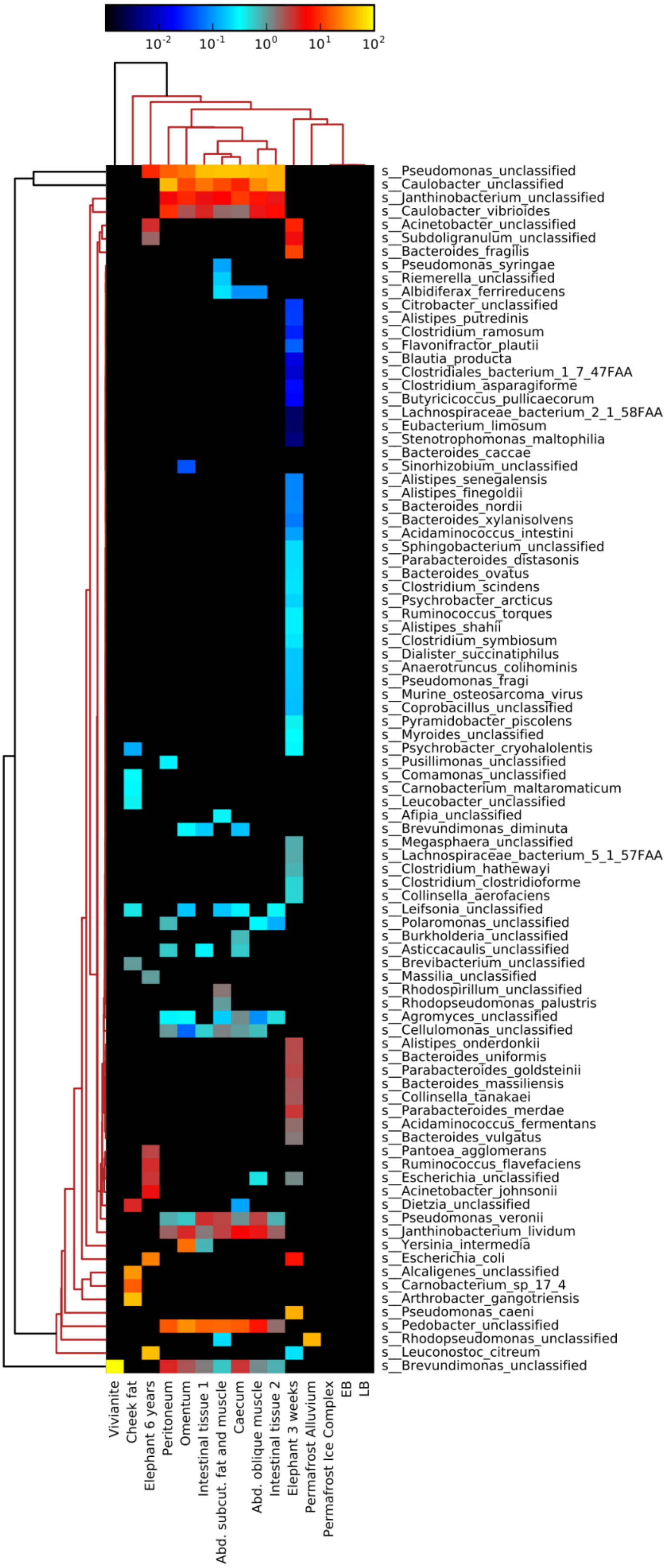

Figure S6. Heat map of MetaPhlAn2 results based on Euclidean distances.
